# Artificial oocyte activation may improve embryo quality in older patients with diminished ovarian reserve undergoing IVF-ICSI cycles

**DOI:** 10.1186/s13048-022-01036-7

**Published:** 2022-09-09

**Authors:** Tzung-En Tsai, Pei-Hsuan Lin, Pei-Fen Lian, Chia-Jung Li, Salvatore Giovanni Vitale, Mislav Mikuš, Wan-Ping Su, Hsiao-Wen Tsai, Kuan-Hao Tsui, Li-Te Lin

**Affiliations:** 1 Department of Obstetrics and Gynecology, Kaohsiung Veterans General Hospital, No.386, Dazhong 1st Rd., Zuoying Dist, 81362 Kaohsiung City, Taiwan; 2 Institute of Biopharmaceutical Sciences, National Sun Yat-sen University, Kaohsiung City, Taiwan; 3 Obstetrics and Gynecology Unit, Department of General Surgery and Medical Surgical Specialties, University of Catania, 95124 Catania, Italy; 4 Department of Obstetrics and Gynecology, University Hospital Centre Zagreb, 10000 Zagreb, Croatia; 5 Department of Obstetrics and Gynecology, School of Medicine, National Yang-Ming University, Taipei City, Taiwan; 6 Department of Biological Science, National Sun Yat-sen University, Kaohsiung City, Taiwan

**Keywords:** Artificial oocyte activation, Calcium ionophore, In vitro fertilization, Intracytoplasmic sperm injection, Embryo development, Embryo quality

## Abstract

**Background:**

Artificial oocyte activation (AOA) is used to improve fertilization rate following fertilization failure after intracytoplasmic sperm injection (ICSI). Several studies have also shown that AOA may be involved in embryo development. Women with poor ovarian response are more likely to encounter in vitro fertilization (IVF) failure due to poor embryo quality. The aim of this study was to investigate whether AOA could improve embryo quality in older patients with diminished ovarian reserve undergoing IVF-ICSI cycles.

**Methods:**

The retrospective cohort study consisted of 308 patients who fulfilled the POSEIDON Group 4 criteria and received IVF-ICSI cycles. The study group included 91 patients receiving AOA with calcium ionophores following ICSI. A total of 168 patients in the control group underwent ICSI without AOA. The baseline and cycle characteristics and embryo quality were compared between the two groups.

**Results:**

At baseline, there were more IVF attempts, greater primary infertility, higher basal FSH levels and lower anti-Müllerian hormone (AMH) levels in the AOA group than in the non-AOA group. In terms of embryo quality, there were higher cleavage rates and top-quality Day 3 embryo (TQE) rates, as well as higher percentages of more than 1 TQE and TQE rates ≥50 in the AOA group than in the non-AOA group. The multivariate analysis revealed that AOA was positively associated with more than 1 TQE (adjusted OR 3.24, 95% CI 1.63–6.45, *P* = 0.001) and a TQE rate ≥ 50 (adjusted OR 2.14, 95% CI 1.20–3.80, *P* = 0.010). When the study population was divided into 2 subgroups based on the age of 40 years old, the beneficial effects of AOA on embryo quality were only observed in the subgroup of age ≥ 40 years old.

**Conclusions:**

Our data suggest that AOA with calcium ionophores may improve embryo quality in older patients with diminished ovarian reserve undergoing IVF-ICSI cycles, especially in women aged ≥40 years.

## Introduction

Intracytoplasmic sperm injection (ICSI) is a procedure involving the injection of one spermatozoon into the cytoplasm of an oocyte to improve fertilization and pregnancy in couples with male factor infertility [1]. The fertilization rate may reach 70% by ICSI. However, complete fertilization failure or an extremely low fertilization rate occurs in approximately 1 ~ 5% of ICSI cycles [2]. Fertilization failure is considered to be related to a deficiency of oocyte activation, which may be related to sperm or oocyte factors [3]. There are a series of complex molecular events related to oocyte activation that originate from the entry of sperm and result in oscillations from intracellular calcium released from the endoplasmic reticulum [4–6].

Artificial oocyte activation (AOA) is a way to artificially induce calcium release following ICSI and can be performed in a variety of ways, such as through chemical, mechanical or physical stimuli [7]. Calcium ionophores such as ionomycin and calcimycin are chemical agents most commonly used to induce oocyte activation [2, 3]. These chemical agents enhance intracellular calcium release from endoplasmic reticulum and facilitate the influx of extracellular calcium [8]. AOA is mainly used in patients with fertilization failure or a lower fertilization rate after ICSI, and several studies have demonstrated that AOA indeed improves the fertilization rate in these patients [9–11]. In addition, other studies have shown that increases in calcium signaling are an essential factor to induce both nuclear and cytoplasmic changes in fertilized oocytes and lead to not only oocyte activation but also early onset of embryogenesis [7, 8]. Furthermore, there are studies that suggest that AOA might improve embryo development [6, 12, 13].

Poor ovarian responders (PORs) are a group of women who have a poor response to ovarian stimulation during IVF cycles. Treating PORs is a great challenge due to their poor IVF outcomes [14]. As previously described, the definition of PORs varies widely in previous studies [14]. In 2016, the Patient-Oriented Strategies Encompassing Individualized Oocyte Number (POSEIDON) group proposed a new classification for patients with low prognosis to advocate an individualized approach [15]. POSEIDON Group 4 refers to women with advanced age and diminished ovarian reserve who usually have poor embryo quality and impaired IVF outcomes [16, 17]. The numbers of POSEIDON Group 4 patients are increasing and are more than half of the total population of the POSEIDON group [18, 19].

Taken together, we hypothesized that AOA could enhance embryo quality in PORs. Few studies have been published to explore this issue. Thus, a retrospective cohort study was designed to investigate the effects of AOA on embryo quality in patients who fulfilled POSEIDON Group 4 criteria and received IVF-ICSI treatment.

## Materials and methods

### Study design and participants

**Fig. 1:**
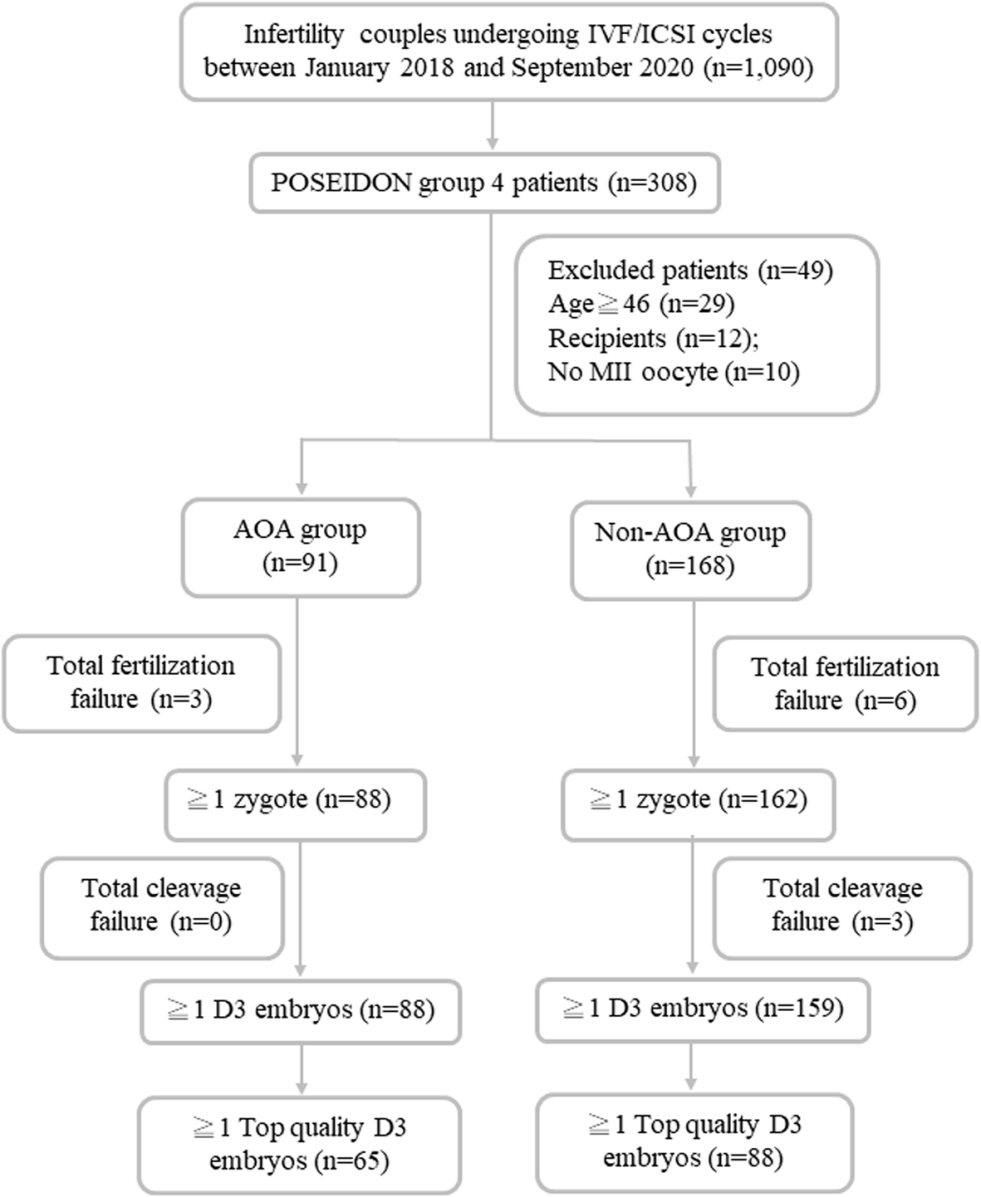
Study flow chart

The retrospective cohort study was performed at the reproductive medical center of Kaohsiung Veterans General Hospital from January 2018 to September 2020. The study project was approved by the institutional review board at Kaohsiung Veterans General Hospital (reference number of institutional review board: KSVGH21-CT1–27). The requirement for consent was waived by the institutional review board because of its retrospective design. All patient data were collected from electronic medical records and IVF treatment sheets. Patients who received the first IVF-ICSI cycle in our reproductive medical center and fulfilled the criteria of POSEIDON Group 4 [age ≥ 35 years old, with antral follicle counts (AFCs) < 5 and/or anti-Müllerian hormone (AMH) < 1.2 ng/ml] were included in this study. A total of 308 patients met the POSEIDON Group 4 criteria. The exclusion criteria were as follows: (1) patients whose age was over 46 years old, (2) patients who were oocytes recipients, and (3) patients who obtained no metaphase II oocytes. Eventually, 259 POSEIDON Group 4 patients undergoing IVF-ICSI cycles were identified and divided into the AOA group (*n* = 91) and the non-AOA group (*n* = 168). A calcium ionophore was used to activate oocytes following the ICSI procedure in the AOA group but not in the non-AOA group. The study flow chart is shown in Fig. 1.

### Treatment protocols

The stimulation protocols in the current study included the GnRH antagonist protocol, ultralong protocol, and progestin priming ovulation stimulation (PPOS) protocol. For the GnRH antagonist protocol, within 5 days of the menstrual cycle, controlled ovarian stimulation was initiated with a gonadotropin, such as recombinant follicle-stimulating hormone (rFSH) plus recombinant luteinizing hormone (Pergoveris, Merck Serono SA, Aubonne, Switzerland) or human menopausal gonadotropin (Merional, IBSA Institut Biochimique S.A., Lamone, Switzerland) after transvaginal ultrasound evaluation. GnRH antagonists (Cetrotide 0.25 mg, Pierre Fabre Medicament Production, Aquitaine Pharm International, Idron, France) were injected daily when the leading follicle reached a diameter of 12–14 mm. Once the leading follicle reached a diameter of 18 mm, final oocyte maturation was triggered by a dual trigger comprising recombinant human chorionic gonadotropin (rhCG, Ovidrel 250 μg, Merck Serono S.p.A., Modugno, Italy) and GnRH agonist (Lupro 2 mg, Nang Kuang Pharmaceutical Co, Ltd., Tainan, Taiwan). Oocyte retrieval with the guidance of transvaginal ultrasound was performed 36 hours later. For the ultralong protocol, a long-acting GnRH agonist (Leuplin Depot 3.75 mg S. C, Takeda Pharmaceutical Company Limited, Yodogawa-ku, Osaka, Japan) was pretreated in the previous menstrual cycle. Controlled ovarian stimulation with gonadotropins as described above started approximately 4 weeks later. When the leading follicle reached a diameter of 18 mm, rhCG was used for oocyte triggering. Then, ultrasound-guided oocyte aspiration was conducted 36 hours later. In patients receiving the PPOS protocol, after evaluation of transvaginal ultrasound, controlled ovarian stimulation was started within 5 days of the menstrual cycle. Gonadotropins such as rFSH plus recombinant luteinizing hormone (Pergoveris, Merck Serono SA, Aubonne, Switzerland) or human menopausal gonadotropin (Merional, IBSA Institut Biochimique S.A., Lamone, Switzerland or Menopur, Ferring International Center SA, Saint-Prex, Switzerland) combined with oral medicines including 100 mg clomiphene citrate (Clomiphene tablets 50 mg, Yung Shin Pharmaceutical Industrial Co. LTD., Taichung, Taiwan) once per day and 10 mg medroxyprogesterone acetate (Provera 5 mg, Pfizer Italia S.r.l, Ascoli Piceno, Italy) twice daily were administered. While the leading follicle reached a diameter of 18 mm, final oocyte maturation was induced by a dual trigger. Oocyte retrieval guided by transvaginal ultrasound was arranged 36 hours later.

### Artificial oocyte activation

Approximately 4 hours after oocyte retrieval, fertilization with ICSI was performed. As previous studies [20, 21], in the AOA group, the calcium ionophore (GM508 CultActive, GYNEMED GmbH & Co. KG, Germany) was incubated at 37 °C in 6% CO2 for 4 hours before application. The injected oocytes were incubated in 50 μl pre-equilibrated calcium ionophore drops individually for 15 min immediately after ICSI. Then, the injected oocytes were removed from the drop and washed twice in HEPES- or MOPS-free medium. After washing, the injected oocytes were transferred to the culture medium for further culture.

### Embryo culture and assessment

Embryos were cultured using a microdrop at 37 °C in a 6% or 6.5% CO2 incubator. Embryo quality was assessed based on the criteria described by the Istanbul consensus workshop [22]. Embryos were graded on Day 3 after oocyte retrieval as Grade 1 to Grade 3 based on the percentage of fragmentation, the evenness of each blastomere and whether multinucleation was present. In brief, Grade 1 embryos had less than 10% fragmentation, equal-sized blastomeres and no multinucleation; Grade 2 embryos had 10–25% fragmentation, equal-sized blastomeres in the majority of cells and no multinucleation; and Grade 3 embryos had more than 25% fragmentation, blastomeres of unequal size and evidence of multinucleation. Top-quality Day 3 embryos (TQE) were defined as 6–10 cells with grade 1 (< 10% fragmentation, equal-sized blastomeres and no multinucleation) in this study. A freeze-all policy was adopted in our reproductive medical center. Because of poor embryo quality in the study population, most of the embryos were cryopreserved in the cleavage stage, and few embryos were extendedly cultured to the blastocyst stage.

### Outcome measures

The primary outcomes were cleavage rate and top-quality Day 3 embryo rate. The secondary outcomes included numbers of Day 3 embryos and numbers of top-quality Day 3 embryos.

The cleavage rate was defined as the number of Day 3 embryos divided by the number of fertilized oocytes. The top-quality Day 3 embryo rate was defined as the number of top-quality Day 3 embryos divided by the number of Day 3 embryos.

### Statistical analysis

The Kolmogorov–Smirnov test was used to test the normal distribution of continuous variables. A Student’s t test or the Mann–Whitney U test was used to evaluate quantitative variables. A chi-square test was used to assess categorical variables. Logistic regression was used to calculate the odds ratio and 95% confidence intervals (CI) of the two groups (AOA and non-AOA). All data analyses were conducted using the Statistical Package for Social Sciences (SPSS) version 20.0 (Chicago, IL, USA). A two-tailed value of *P* <  0.05 was considered statistically significant.

## Results

As shown in Fig. 1, there were 1090 IVF/ICSI cycles from January 2018 until September 2020. Out of the 1090 cycles, 308 patients met the criteria of POSEIDON Group 4 and received the first IVF-ICSI cycle in our reproductive medical center. Among the 308 patients, there were 29 cases in which the patients were older than 46 years old, 12 cases in which patients were oocytes recipients, and 10 cases in which there were no metaphase II oocytes. Therefore, 49 cycles were excluded. The remaining 259 patients were included and divided into the AOA group (*n* = 91) and the non-AOA group (*n* = 168). Next, 3 patients with total fertilization failure were noted in the AOA group, and 6 patients with total fertilization failure were observed in the non-AOA group. Finally, the AOA group included 88 patients with viable zygotes, and the non-AOA group included 162 patients with viable zygotes for further evaluation of embryo quality.

The baseline characteristics of the study population are summarized in Table 1. No significant differences regarding age, body mass index, infertility duration or AFCs were noted between the two groups. However, more previous IVF attempts, more primary infertility, higher basal FSH levels and lower AMH levels were observed in the AOA group than in the non-AOA group.

**Table 1:** Baseline characteristics of POSEIDON Group 4 patients with AOA or non-AOA

Parameters	AOA (*n* = 91)	Non-AOA (*n* = 168)	*P* value
Age (years)	40.9 ± 2.8	40.4 ± 2.7	0.138
Body mass index (kg/m^2^)	23.1 ± 3.8	23.4 ± 3.5	0.540
Infertility duration (years)	5.0 ± 3.5	4.6 ± 3.7	0.478
Previous IVF attempts (%)			0.009
0	20.9%(19/91)	39.3%(66/168)	
1 ~ 2	42.9%(39/91)	35.7%(60/168)	
≧3	36.3%(33/91)	25.0%(42/168)	
Types of infertility (%)			0.033
Primary infertility	62.6%(57/91)	48.8%(82/168)	
Secondary infertility	37.4%(32/91)	51.2%(86/168)	
Basal FSH (IU/l)	8.5 ± 9.3	5.4 ± 3.8	0.003
Antral follicle counts (n)	6.3 ± 3.0	6.9 ± 3.6	0.156
Anti-Müllerian hormone (ng/mL)	0.57 ± 0.32	0.67 ± 0.32	0.028

Data are presented as the mean ± standard deviation and %

*AOA* Artificial oocyte activation, *IVF* In vitro fertilization, *FSH* Follicle-stimulating hormone

As shown in Table 2, there were no significant differences in stimulation duration, gonadotropin dosage, stimulation protocols, number of oocytes retrieved, number of metaphase II oocytes, maturation rate, number of fertilized oocytes, fertilization rate, total fertilization failure rate, number of Day 3 embryos or number of top-quality Day 3 embryos. However, patients in the AOA group had a higher cleavage rate (94.9 ± 28.0% vs. 86.5 ± 22.8%, *P* = 0.018) and top-quality Day 3 embryo (TQE) rate (53.2 ± 39.1% vs. 37.2 ± 42.7%, *P* = 0.004) than those in the non-AOA group. Moreover, the percentage of more than 1 TQE (73.9% vs. 55.6%, *P* = 0.005) and TQE rate ≥ 50 (60.2% vs. 42.5%, *P* = 0.008) were higher in the AOA group than in the non-AOA group.

**Table 2:** Cycle characteristics of POSEIDON Group 4 patients with AOA or non-AOA

Parameters	AOA (*n* = 91)	Non-AOA (*n* = 168)	*P* value
Stimulation duration (days)	11.1 ± 2.2	11.0 ± 2.1	0.684
Gonadotropin dosage (IU)	3132.4 ± 701.0	3132.4 ± 697.9	1.000
Stimulation protocols (%)			0.807
GnRH antagonist protocol	60.4%(55/91)	64.3%(108/168)	
PPOS protocol	27.5%(25/91)	25.6%(43/168)	
Ultralong protocol	12.1%(11/91)	10.1%(17/168)	
No. of oocytes retrieved (n)	4.1 ± 2.6	4.5 ± 3.0	0.329
No. of metaphase II oocytes (n)	3.2 ± 2.1	3.6 ± 2.5	0.132
Maturation rate (%)	80.7 ± 20.8	82.1 ± 19.3	0.581
No. of fertilized oocytes (n)	2.7 ± 1.8	3.0 ± 2.1	0.258
Fertilization rate (%)	86.2 ± 24.1	83.0 ± 26.0	0.340
Total fertilization failure rate (%)	3.3%	4.2%	0.715
No. of Day 3 embryos (n)	2.6 ± 1.6	2.8 ± 2.1	0.443
Cleavage rate (%)	94.9 ± 28.0	86.5 ± 22.8	0.018
No. of top-quality Day 3 embryos (n)	1.4 ± 1.3	1.2 ± 1.5	0.414
≧1 top-quality Day 3 embryos	73.9%	55.6%	0.005
Top-quality Day 3 embryos rate (%)	53.2 ± 39.1	37.2 ± 42.7	0.004
Top-quality Day 3 embryos rate≧50	60.2%	42.5%	0.008

Data are presented as the mean ± standard deviation and %

*AOA* Artificial oocyte activation, *GnRH* Gonadotropin releasing Hormone, *PPOS* Progestin priming ovulation stimulation

In Table 3, a binary logistic regression analysis was performed to analyze whether using AOA would affect TQE. Confounding parameters such as age, body mass index, previous IVF attempts, types of infertility, basal FSH, AFCs, AMH and number of metaphase II oocytes were included in the analysis. The multivariate analysis revealed that AOA positively correlated with more than 1 TQE (adjusted OR 3.24, 95% CI 1.63–6.45, *P* = 0.001) and TQE rate ≥ 50 (adjusted OR 2.14, 95% CI 1.20–3.80, *P* = 0.010). The number of metaphase II oocytes was also positively related to more than 1 TQE (adjusted OR 2.19, 95% CI 1.65–2.91, *P* <  0.001) and TQE rate ≥ 50 (adjusted OR 1.20, 95% CI 1.03–1.41, *P* = 0.024).

**Table 3:** Analyses of factors affecting top-quality Day 3 embryos (TQE) in POSEIDON Group 4 patients using logistic regression

	≧1 TQE		TQE rate≧50	
	Adjusted OR (95% CI)	*P* value	Adjusted OR (95% CI)	*P* value
AOA vs. non-AOA	3.24 (1.63–6.45)	0.001	2.14 (1.20–3.80)	0.010
Age (years)	1.05 (0.94–1.18)	0.386	1.02 (0.93–1.13)	0.658
BMI (kg/m^2^)	1.01 (0.92–1.10)	0.845	0.97 (0.90–1.05)	0.496
Previous IVF attempts	0.83 (0.55–1.25)	0.361	0.80 (0.56–1.12)	0.193
Types of infertility	0.70 (0.36–1.33)	0.269	0.85 (0.49–1.47)	0.552
Basal FSH (IU/l)	1.03 (0.97–1.09)	0.378	1.03 (0.97–1.08)	0.364
AFC (n)	1.00 (0.88–1.13)	0.943	0.99 (0.89–1.11)	0.891
AMH (ng/mL)	0.47 (0.16–1.41)	0.179	0.82 (0.32–2.07)	0.670
No. of MII oocytes (n)	2.19 (1.65–2.91)	< 0.001	1.20 (1.03–1.41)	0.024

*OR* Odds ratio, *CI* Confidence interval, *AOA* Artificial oocyte activation, *BMI* Body mass index, *IVF* in vitro fertilization, *FSH* Follicle-stimulating hormone, *AFC* Antral follicle count, *AMH* Anti-Müllerian hormone, *MII* Metaphase II

We then divided our study population into 2 subgroups based on the age of 40 years old (Table 4). In the subgroup of age ≥ 40 years old, compared to the non-AOA group, the AOA group obtained a significantly higher cleavage rate (96.3 ± 31.3% vs. 86.6 ± 24.3%, *P* = 0.035) and TQE rate (56.8 ± 37.7% vs. 34.6 ± 37.7%, *P* = 0.001) as well as a higher percentage of more than 1 TQE (78.9% vs. 53.7%, *P* = 0.002) and TQE rate ≥ 50 (64.9% vs. 41.1%, *P* = 0.004). However, in the subgroup of age < 40 years old, cleavage rate, TQE rate, percentage of more than 1 TQE and percentage of TQE ≥ 50 were similar between the two groups.

**Table 4:** Subgroup analyses (categorized relative to the age of 40 years) of POSEIDIN Group 4 patients with AOA or non-AOA

	< 40 (35 ~ 39) y/o			≧ 40 (40 ~ 45) y/o		
Parameters	AOA (*n* = 32)	Non-AOA (*n* = 68)	*P* value	AOA (*n* = 59)	Non-AOA (*n* = 100)	*P* value
Age (years)	37.8 ± 1.5	37.5 ± 1.3	0.378	42.6 ± 1.6	42.3 ± 1.4	0.240
Body mass index (kg/m^2^)	22.4 ± 3.0	23.1 ± 3.0	0.263	23.5 ± 4.2	23.5 ± 3.7	0.889
Antral follicle counts (n)	5.9 ± 2.6	7.8 ± 3.6	0.012	6.5 ± 3.2	6.3 ± 3.5	0.794
Anti-Müllerian hormone (ng/ml)	0.50 ± 0.30	0.72 ± 0.29	0.001	0.61 ± 0.33	0.63 ± 0.34	0.728
No. of oocytes retrieved (n)	3.3 ± 2.1	5.0 ± 3.1	0.008	4.5 ± 2.8	4.1 ± 2.9	0.394
No. of metaphase II oocytes (n)	2.6 ± 1.8	4.0 ± 2.5	0.007	3.5 ± 2.2	3.4 ± 2.4	0.833
Maturation rate (%)	81.3 ± 21.8	82.3 ± 17.6	0.808	80.4 ± 20.4	82.0 ± 20.5	0.626
No. of fertilized oocytes (n)	2.3 ± 1.7	3.4 ± 2.3	0.024	3.0 ± 1.9	2.8 ± 2.0	0.618
Fertilization rate (%)	86.9 ± 23.5	82.5 ± 24.8	0.403	85.8 ± 24.6	83.4 ± 26.8	0.575
No. of Day 3 embryos (n)	2.2 ± 1.5	3.0 ± 2.3	0.084	2.8 ± 1.6	2.6 ± 2.0	0.547
Cleavage rate (%)	92.2 ± 20.8	86.3 ± 20.6	0.200	96.3 ± 31.3	86.6 ± 24.3	0.035
No. of top-quality Day 3 embryos (n)	1.2 ± 1.4	1.3 ± 1.6	0.604	1.5 ± 1.2	1.2 ± 1.4	0.120
≧1 top-quality Day 3 embryos	64.5%	58.5%	0.571	78.9%	53.7%	0.002
Top-quality Day 3 embryos rate (%)	46.5 ± 41.5	37.1 ± 37.0	0.267	56.8 ± 37.7	34.6 ± 37.7	0.001
Top-quality Day 3 embryos rate≧50	51.6%	44.6%	0.521	64.9%	41.1%	0.004

Data are presented as the mean ± standard deviation and % (n)

*AOA* Artificial oocyte activation

As presented in Table 5, a binary logistic regression analysis was conducted to analyze the effects of AOA on TQE in the subgroup of age ≥ 40 years old. Age, body mass index, previous IVF attempts, types of infertility, basal FSH, AFCs, AMH and number of metaphase II oocytes were considered confounding factors in this analysis. The multivariate analysis revealed that AOA positively correlated with more than 1 TQE (adjusted OR 3.83, 95% CI 1.58–9.29, *P* = 0.003) and TQE rate ≥ 50 (adjusted OR 2.79, 95% CI 1.34–5.82, *P* = 0.006). Furthermore, the number of metaphase II oocytes was positively related to more than 1 TQE (adjusted OR 2.02, 95% CI 1.42–2.89, *P* < 0.001).

**Table 5:** Analyses of factors affecting top-quality Day 3 embryos (TQE) in POSEIDON Group 4 patients aged ≥40 years using logistic regression

	≧1 TQE		TQE rate≧50	
	Adjusted OR (95% CI)	*P* value	Adjusted OR (95% CI)	*P* value
AOA vs. non-AOA	3.83 (1.58–9.29)	0.003	2.79 (1.34–5.82)	0.006
Age (years)	0.99 (0.75–1.30)	0.923	0.92 (0.73–1.17)	0.493
BMI (kg/m^2^)	0.96 (0.87–1.07)	0.475	0.94 (0.86–1.03)	0.153
Previous IVF attempts	0.77 (0.45–1.34)	0.361	0.79 (0.50–1.24)	0.303
Types of infertility	0.76 (0.33–1.76)	0.524	0.92 (0.45–1.88)	0.818
Basal FSH (IU/l)	1.04 (0.95–1.15)	0.391	1.04 (0.96–1.12)	0.394
AFC (n)	1.01 (0.86–1.20)	0.873	0.98 (0.86–1.13)	0.811
AMH (ng/mL)	0.32 (0.08–1.23)	0.098	0.56 (0.18–1.78)	0.329
No. of MII oocytes (n)	2.02 (1.42–2.89)	< 0.001	1.18 (0.96–1.44)	0.119

*OR* Odds ratio, *CI* Confidence interval, *AOA* Artificial oocyte activation, *BMI* Body mass index, *IVF* in vitro fertilization, *FSH* Follicle-stimulating hormone, *AFC* Antral follicle count, *AMH* Anti-Müllerian hormone, *MII* Metaphase II

## Discussion

This was a retrospective study focusing on the effects of AOA on the quality of embryos in patients who fulfilled the POSIDEN Group 4 criteria and underwent IVF-ICSI cycles. This study showed that AOA with calcium ionophores were associated with a higher cleavage rate and TQE rate. In addition, the multivariate analysis revealed a 3.24-fold increase in the possibility of more than 1 TQE (95% CI 1.63–6.45, P = 0.001) and a 2.14-fold increase in the possibility of a TQE rate ≥ 50 (95% CI 1.20–3.80, P = 0.001) in the POSEIDON Group 4 patients with AOA compared to those with non-AOA.

AOA with calcium ionophores can activate oocytes by increasing the permeability of calcium in the cell membrane and inducing the entry of extracellular calcium into the cell, leading to calcium oscillations [23]. Normal calcium oscillations induced by the specific sperm protein phospholipase C zeta (PLCζ) may also play a role in the development of embryos [24–26]. Calcium oscillations stimulate the processes that are required in embryonic development, such as mitotic cleavage, in numerous species of invertebrates and vertebrates [27]. Calcium signals mediate the cell cycle of early embryogenesis. Embryos undergo a series of rapid cell divisions after fertilization [28]. The different frequencies and patterns of calcium oscillations in specific species could affect the induction of oocyte activation and embryo development [29]. The frequency of calcium oscillations was related to different morphogenesis of early embryonic stages. A certain range of the frequency of calcium oscillations is optimal for embryogenesis. Calcium oscillations that are too frequent and too rare are harmful to the embryos [30]. Meiosis and embryonic divisions are regulated by the pattern of calcium oscillations, and a certain threshold of calcium oscillations is essential to induce the formation of pronuclei [31, 32]. The concentration of PLCζ is positively related to the frequency of calcium oscillations. A higher concentration of PLCζ results in a higher frequency of calcium oscillations, which will cause cleavage stage arrest, while a appropriate concentration of PLCζ leads to a appropriate frequency of calcium oscillations, which will activate embryonic development to the blastocyst stage [33, 34]. The calcium-binding proteins in the human endoplasmic reticulum and intracellular calcium channels that regulate inositol 1,4,5-trisphosphate receptors (IP3Rs) play an important role in the regulation of calcium signaling during oocyte maturation, fertilization and early embryo development as calcium signaling is a key factor in early embryonic cleavage [35, 36]. Taken together, calcium oscillations may be an essential element of early embryonic division [37]. Therefore, AOA with calcium ionophores, which could enhance calcium oscillations, may have beneficial effects on embryo development.

The abovementioned studies support our results that AOA with calcium ionophores are positively associated with high embryo quality. In a randomized, open-label trial proposed by Mohamed Fawzy et al., 343 participants, 18–40 years of age, were divided into 3 groups: AOA with SrCl2 following ICSI (*n* = 115), AOA with calcimycin after ICSI (*n* = 113) and AOA with ICSI alone (n = 115). AOA with SrCl2 following ICSI led to better embryological outcomes, such as top-quality Day 3 embryos (OR 5.16, 95% CI 4.11–6.48, *P* < 0.0001), blastocyst formation rate (OR 1.93, 95% CI 1.60–2.34, P < 0.0001), and high-quality blastocysts (OR 1.85, 95% CI 1.51–2.26, P < 0.0001), compared to ICSI alone. Moreover, a higher blastocyst formation rate (OR 1.25, 95% CI 1.03–1.52, *P* = 0.026) was observed in the AOA with calcimycin group after ICSI than in the AOA with ICSI alone group [12]. A meta-analysis conducted by Murugesu and colleagues demonstrated that AOA with calcium ionophores following ICSI significantly improved fertilization, cleavage, blastulation, pregnancy rates and live birth rates. In terms of embryo quality, a better cleavage rate (OR 2.28, 95% CI 1.23–4.21, *P* = 0.06) and blastocyst formation (OR 6.7, 95% CI 2.59–17.28, P = 0.003) were found in the AOA with calcium ionophore group than in the non-AOA group [38]. A prospective study that investigated the use of AOA with calcium ionophores in patients with PLCζ deficiency showed no significant difference between the control (*n* = 113 oocytes), without AOA (*n* = 106 oocytes) and AOA groups (*n* = 114 oocytes) in terms of cleavage rate (91.7 ± 2.8%, 90.9 ± 4.6%, and 95.2 ± 3.4%) and embryo quality score (2.5 ± 0.1, 2.3 ± 0.2, and 2.4 ± 0.2). However, all the female patients enrolled in this study were under the age of 35 years old [39]. In an updated systemic review and meta-analysis, AOA with calcium ionophore treatment significantly improved the blastocyst formation rate (OR 3.59, 95% CI, 1.34–9.60, P = 0.01) but did not improve the cleavage rate (OR 1.68, 95% CI, 0.90–3.13, *P* = 0.10) or top-quality embryo rate (OR 1.15, 95% CI, 0.72–1.82, *P* = 0.55 ) [40]. Thus, more large-scale studies are needed to confirm whether AOA with calcium ionophores can improve embryo quality.

To examine the effects of AOA with calcium ionophores on embryo quality in older patients, the study population was divided into 2 subgroups based on the age of 40 years old. The results showed that AOA with calcium ionophores were associated with a higher cleavage rate and TQE rate only in the subgroup aged ≥40 years old and not in the subgroup aged < 40 years old. In addition, the multivariate analysis revealed that the subgroup of age ≥ 40 years old with AOA had a 3.83-fold increase in the possibility of more than 1 TQE (95% CI 1.58–9.29, *P* = 0.003) and a 2.79-fold increase in the possibility of TQE rate ≥ 50 (95% CI 1.34–5.82, *P* = 0.006) compared to the subgroup of age ≥ 40 years old without AOA. Impaired oocyte maturation, fertilization and embryo development were observed in advanced maternal age [41]. Increased reactive oxygen species (ROS) in aged oocytes change the regulation of the intracellular calcium signal and affect calcium oscillations in fertilized oocytes, which may be associated with poor embryo development [42, 43]. Therefore, AOA with calcium ionophores may have more beneficial effects on older women as these women are more likely to suffer from low intracellular calcium stores and abnormal calcium oscillations. This hypothesis is supported by our result that a higher cleavage rate and TQE rate were only found in the subgroup aged ≥40 years old with AOA rather than in the subgroup aged < 40 years old with AOA. However, more large-scale studies are required to verify our results.

There are some limitations in our study. First, the retrospective design and limited study population are major limitations of this study. Large-scale randomized controlled trials are needed to verify our results. Next, a power calculation is lacking in this study because all patients who met the POSEIDON Group 4 criteria during study period were included. Moreover, the basal characteristics were not similar regarding previous IVF attempts, types of infertility, basal FSH and AMH between the AOA and non-AOA groups. Besides, different IVF protocols, including the antagonist protocol, ultralong protocol, and PPOS protocol, were used in this study, which may be another source of bias. Finally, we should interpret the data cautiously in the subgroup analyses because of the potential bias from the small population. A strength of this study is that all IVF cycles were performed by the same physician and embryologist.

In conclusion, our data suggest that AOA with calcium ionophores may improve embryo quality in POSEIDON Group 4 patients undergoing IVF-ICSI cycles, especially in those older than 40 years old.

## Data Availability

The datasets used and analyzed in this article are available from the corresponding author on reasonable request.

## Abbreviations

AOA: Artificial oocyte activation
IVF: In vitro Fertilization
ICSI: Intracytoplasmic Sperm Injection
POSEIDON Group: Patient-Oriented Strategies Encompassing Individualized Oocyte Number Group
FSH: Follicular stimulating hormone
AMH: Anti-Mullerian Hormone
AFC: Antral follicle counts
GnRH: Gonadotrophin releasing hormone
hCG: human chorionic gonadotropin
PPOS: Progestin priming ovulation stimulation
TQE: Top-quality Day 3 embryos
OR: Odds ratio
PLCζ: Phospholipase C zeta
IP3R: Inositol 1,4,5- trisphosphate receptors
ROS: Reactive oxygen species
ER: Endoplasmic reticulum
RR: Relative risk
CI: Confidence interval
RCT: Randomized controlled trial
